# Establishment and characterization of human pluripotent stem cells-derived brain organoids to model cerebellar diseases

**DOI:** 10.1038/s41598-022-16369-y

**Published:** 2022-07-22

**Authors:** João Brás, Daniel Henriques, Ricardo Moreira, Magda M. Santana, Rita Silva-Pedrosa, Diana Adão, Sandra Braz, Ana Rita Álvaro, Luís Pereira de Almeida, Liliana S. Mendonça

**Affiliations:** 1 CNC-Center for Neurosciences and Cell Biology, University of Coimbra, Coimbra, Portugal; 2 CIBB-Center for Innovative Biomedicine and Biotechnology, University of Coimbra, Coimbra, Portugal; 3 Institute for Interdisciplinary Research, University of Coimbra, Coimbra, Portugal; 4 ICVS-Life and Health Sciences Research Institute, University of Minho, Braga, Portugal; 5 CEB-Center of Biological Engineering, University of Minho, Braga, Portugal; 6 Faculty of Pharmacy, University of Coimbra, Coimbra, Portugal

**Keywords:** Cellular neuroscience, Stem-cell research

## Abstract

The establishment of robust human brain organoids to model cerebellar diseases is essential to study new therapeutic strategies for cerebellum-associated disorders. Machado-Joseph disease (MJD) is a cerebellar hereditary neurodegenerative disease, without therapeutic options able to prevent the disease progression. In the present work, control and MJD induced-pluripotent stem cells were used to establish human brain organoids. These organoids were characterized regarding brain development, cell type composition, and MJD-associated neuropathology markers, to evaluate their value for cerebellar diseases modeling. Our data indicate that the organoids recapitulated, to some extent, aspects of brain development, such as astroglia emerging after neurons and the presence of ventricular-like zones surrounded by glia and neurons that are found only in primate brains. Moreover, the brain organoids presented markers of neural progenitors proliferation, neuronal differentiation, inhibitory and excitatory synapses, and firing neurons. The established brain organoids also exhibited markers of cerebellar neurons progenitors and mature cerebellar neurons. Finally, MJD brain organoids showed higher ventricular-like zone numbers, an indication of lower maturation, and an increased number of ataxin-3-positive aggregates, compared with control organoids. Altogether, our data indicate that the established organoids recapitulate important characteristics of human brain development and exhibit cerebellar features, constituting a resourceful tool for testing therapeutic approaches for cerebellar diseases.

## Introduction

Cerebellar diseases include several conditions such as congenital malformations and hereditary or acquired ataxias^1^. Among those, Machado-Joseph disease (MJD), also known as spinocerebellar ataxia type 3 (SCA3), is the most prevalent autosomal dominant hereditary ataxia with the worldwide prevalence of 0.3–2/100,000^2^, and with the highest prevalence reported worldwide (1/140) at the Flores Island (Azores, Portugal)^1,3^.

MJD is caused by a mutation in the exon 10 of the *MJD1*/*ATXN3* gene, located at the chromosome 14, which leads to an extension of the trinucleotide cytosine-adenine-guanine (CAG), coding for the glutamine aminoacid, resulting in a polyglutamine tract expansion in the ataxin-3 protein^3–5^. The normal ataxin-3 protein has 10–51 glutamines, while the mutant protein carry 55–87 glutamines and tends to form intracellular inclusions. These neuronal intranuclear inclusions are hallmarks of MJD and cause widespread neuronal death within several brain regions, namely in the cerebellum. In addition, the mutant protein aggregates are associated with neuronal dysfunction, such as impaired neuronal communication and synaptic activity or reduced neurite number^3–7^. MJD patients present progressive ataxia and a motor dysfunction that affect gaze, speech, gait, and balance. Depression and cognitive impairments have also been reported in MJD patients^4,6^. Presently, there is no cure or effective treatment to alleviate the symptoms or slow down MJD progression. Therefore, robust MJD brain models might significantly contribute to the development of disease-impacting therapies.

The establishment of human induced pluripotent stem cells (iPSC) from dedifferentiation of specialized cells^8^ represented a major breakthrough for disease modeling. Their potential to differentiate into any cell type of the human body^9^ enable the development of a variety of new in vitro models. Although cellular models are useful and reproducible, when dealing with in vitro disease modeling of brain disorders, it is important to be aware that the human brain is a much more complex three-dimensional (3D) structure. Thus, the traditional two-dimensional cell cultures have strong limitations given that they do not mimic brain tissue complexity, architecture, or physiological environment^10^. On the other hand, given the significant differences between rodents and human brains, not only in size but also in organization and complexity of the human connectome, translation of results obtained with rodents, the most widely used animal models, recurrently fails^10–12^.

Brain organoids in turn, bring together the best of both worlds, they are cell-derived 3D structures with self-organization that reproduce some structural and cellular aspects of brain, i.e. they are in vitro models with some organ-like complexity. These systems are able to reproduce ventricular-like structures with stem cells, the expression of markers of deep and superficial layer neurons, radial glial cells, and neuronal subtypes^10,13,14^. Additionally, human brain organoids mimic some aspects of human cerebral development, including organization of neural progenitor zones and the presence of outer radial glial cells (oRG) in outer subventricular zone (OSVZ) that are only present in primate brains^10,13,14^. Cerebral, cerebellar, and forebrain organoids have been established from human pluripotent stem cells, demonstrating the ability of brain organoids to model human brain^13,15,16^.

In this view, the objective of this work was to evaluate whether a previously established brain organoid protocol modeling whole-brain might also be used to model cerebellar diseases, such as MJD. For that, human brain organoids were established from human iPSC (derived from a control individual and an MJD patient) using a commercially available kit. Then, the established organoids were characterized for brain development and MJD-associated neuropathology markers. Markers of pluripotency (NANOG), neuroectoderm/radial glial cells (PAX6, Nestin), neurons (TUBB3, MAP2), and glial cells (GFAP, S100B) were assessed at different time points of the differentiation. The obtained organoids successfully recapitulated several features of human brain development, such as glia succeeding neurons, the presence of ventricular-like structures, and the presence of oRG in the OSVZ characteristic of primate brains. Moreover, brain organoids presented markers of neural progenitors proliferation (Msi1, Notch1), neuronal differentiation (NeuroD1), neurotrophic factors (BDNF) production, and inhibitory (colocalization of VGAT- and Gephyrin-positive puncta) and excitatory (colocalization of VGlut- and PSD95-positive puncta) synapses. More importantly, the established brain organoids also had markers of cerebellar neuronal progenitors (ATOH1) and cerebellar mature neurons (CALB1, TBR1, and PCP4). Finally, MJD brain organoids with 40 days presented few MJD-associated neuropathology markers, namely no significant presence of mutant ataxin-3 inclusions was detected, an indication that to model late-onset diseases as MJD it might be required longer maturation of the brain organoids. Nevertheless, in MJD brain organoids it was detected that the number of ataxin-3-positive spots/aggregates is higher than in Control organoids, suggesting mutant ataxin-3 protein aggregation. Altogether, data indicate that the established organoids recapitulated important characteristics of human brain development and exhibited cerebellar features, constituting a 3D cell-derived system to model cerebellar disorders, which is of major importance for testing therapeutic approaches.

## Methods

### Ethics approval and informed consent

Informed consent was obtained from all participants. The isolation of human fibroblasts for the iPSC derivation was performed at the Hospital Center of the University of Coimbra under informed consent and approved by the Ethics Committee of the Medical Faculty of the University of Coimbra, Portugal, and followed the protocols and guidelines also approved by this Ethics Committee.

### Induced-pluripotent stem cells (iPSC) culture

Human iPSC were obtained by reprogramming dermal fibroblasts from a control and an MJD patient into iPSC by forced expression of reprogramming factors (Oct-4, Klf4, c-Myc, and Sox-2), as previously described^8,17^, mediated by lentivirus. The used fibroblasts from one MJD patient (79 CAG repeats) and one control (23 CAG repeats) individual were previously collected and its karyotype and genotype analysis, demonstrating a normal diploid karyotype and number of CAG repeats respectively, was previously showed^18^.

IPSC were grown using mTeSR1 Complete Kit GMP (STEMCELL technologies), supplemented with 1% Penicillin/Streptomycin (Invitrogen) and kept at 37ºC with 5% CO_2_. The cells were cultured as monolayers in Matrigel (hESC-qualified Matrix LDEV-Free, BD Matrigel™, Corning) coated plates and passed every 5–7 days using Versene (Gibco) as a non-enzymatic cell dissociation reagent.

### Brain organoids culture

**Figure 1:**
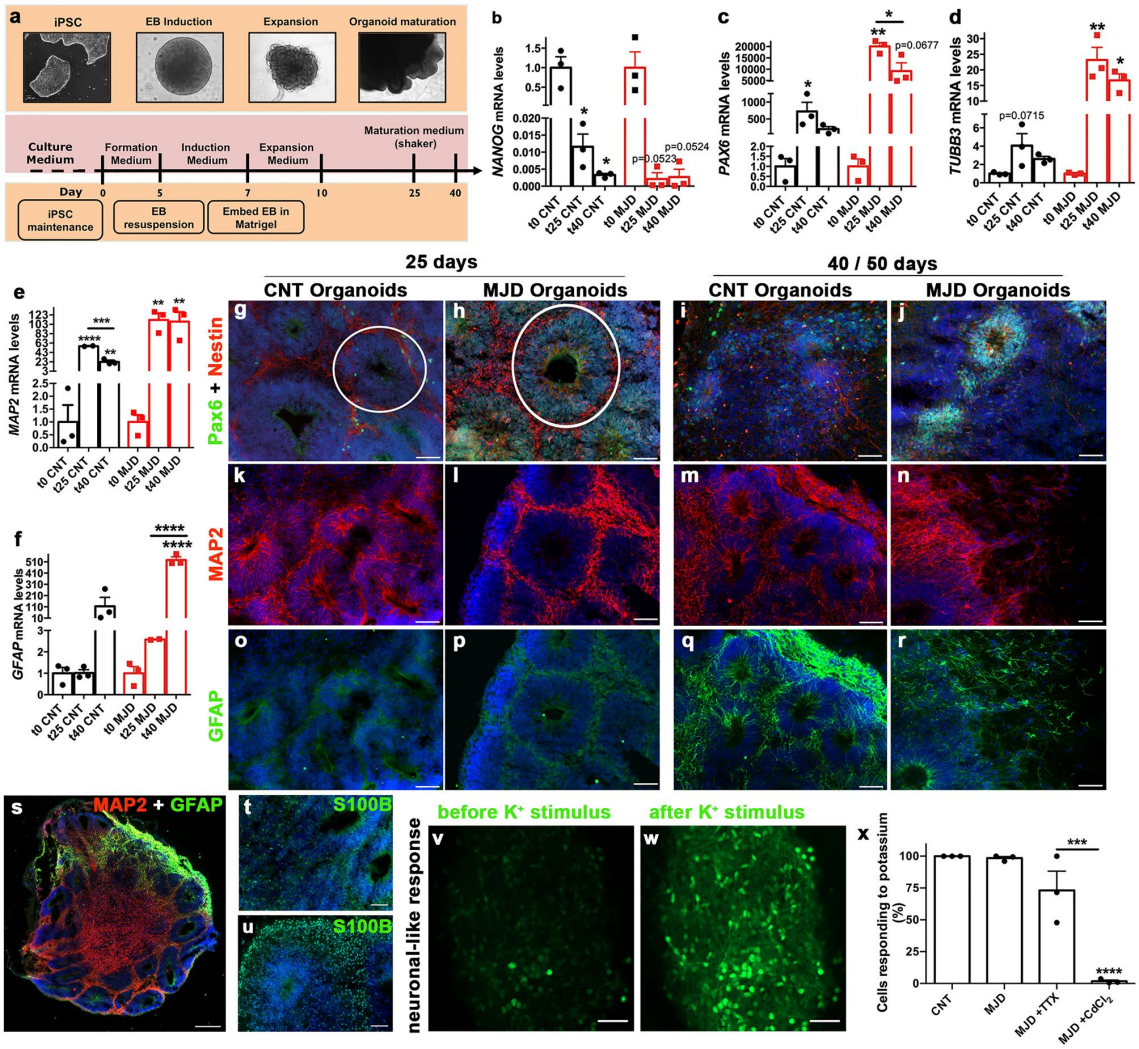
Control and MJD brain organoids present ventricular-like zones, neural progenitor cells, glia, and neurons. **(a)** Schematic representation of the brain organoid protocol used, *iPSC* induced pluripotent stem cells, *EB* embryoid body. **(b–f)** *NANOG*, *PAX6*, *TUBB3*, *MAP2*, and *GFAP* mRNA levels evaluation through RT-qPCR of control (CNT) and MJD organoids at different time points (day 0, 25, and 40, respectively t0, t25, and t40) normalized for the respective t0. Data indicated that *NANOG* mRNA levels, a marker of pluripotency, decreases over time and the mRNA levels of neuronal (*TUBB3* and *MAP2*), glia (*GFAP*), and neuroectoderm (*PAX6*) increase. **(g–u)** Immunohistochemistry evaluation of CNT and MJD organoids with 25 and 40/50 days for **(g–j)** Pax6 (green) and Nestin (red), (**k–n**) MAP2 (red) and **(o-r)** GFAP (green) expression, demonstrating the presence of these markers; white circles: ventricular-like zone. **(s)** MAP2 and GFAP expression and nuclear staining (DAPI: blue) revealing the presence of neuronal and glial cells surrounding ventricular regions in control organoids with 40 days. **(t–u)** S100B (green) expression in (**t**) control and (**u**) MJD organoids with 40/50 days. **(v–x)** Single-cell calcium imaging assay showing the presence of potassium firing neurons in CNT and MJD organoids with 35 days. Representative images of CNT brain organoids **(v)** before and **(w)** after potassium (K +) stimulus administration. **(x)** Quantification of cells responding to potassium stimulus in CNT and MJD organoids, and in MJD organoids upon inhibition of voltage-gated sodium channels with TTX (MJD + TTX) and inhibition of voltage-gated calcium channels with CdCl_2_ (MJD + CdCl_2_). Data are expressed as mean ± SEM (n: 3 independent experiments), *p < 0.05, **p < 0.01, ***p < 0.001, ****p < 0.0001 (**b–f**: comparison established with the correspondent t0 or between the conditions indicated by the lines), one-way ANOVA followed by Tukey posttest. DAPI: blue, representative images of 3 independent experiments, scale bars: 50 μm and (s) 200 μm.

Whole-brain organoids were generated with the STEMdiff™ Cerebral Organoid Kit (STEMCELL technology) as depicted in Fig. 1a. At day 0, iPSC colonies were dissociated into single cells with Versene (Gibco). A total of 9000 cells/well were plated in ultra-low attachment 96-well plates (Corning Costar) and maintained in 100 µL of EB Seeding Medium (STEMdiff™ Cerebral Organoid Basal Medium 1, supplemented with STEMdiff™ Cerebral Organoid Supplement A, and 10 µM Y-27632). At day 2 and 4, 100 µL of EB Formation medium (STEMdiff™ Cerebral Organoid Basal Medium 1 supplemented with STEMdiff™ Cerebral Organoid Supplement A) was added to the culture. At day 5, embryoid bodies were transferred to a 24-well ultra-low attachment plate (Corning Costar) and cultured in Induction Medium (STEMdiff™ Cerebral Organoid Basal Medium 1 and STEMdiff™ Cerebral Organoid Supplement B). At day 7, embryoid bodies were transferred into cold Matrigel (hESC-qualified Matrix LDEV-Free, BD Matrigel™, Corning) droplets, and subsequently incubated at 37ºC with 5% CO_2_ for 30 min to induce Matrigel polymerization. Then, the Matrigel droplets embedding the embryoid bodies were incubated in Expansion Medium (STEMdiff™ Cerebral Organoid Basal Medium 2 with STEMdiff™ Cerebral Organoid Supplement C and STEMdiff™ Cerebral Organoid Supplement D) in a 6-well ultra-low attachment plate (Corning Costar). At day 10, organoids were placed in maturation medium (STEMdiff™ Cerebral Organoid Basal Medium 2 and STEMdiff™ Cerebral Organoid Supplement E in a 49:1 ratio) in an incubator with orbital shaker (65 rpm), at 37ºC with 5% CO_2_, and kept in these conditions until the end of the experiment.

### Organoids processing and immunohistochemistry

Organoids were collected at different time points (day 25, 40, 50, and 120), washed three times in Phosphate-buffered saline (PBS) and incubated in cold 4% paraformaldehyde (PFA) for 20 min at 4 ºC. Organoids were washed again three times in PBS, kept in 30% sucrose in PBS solution for 24 h at 4 ºC, and then were frozen at −80 ºC in Tissue-Tek O.C.T.™ Compound (Sakura) until further sectioning. Organoids were sliced at 20 µm using a cryostat (Thermo Fisher Scientific) and collected in Superfrost™ Plus slides (Thermo Scientific). Slides were dried at 37 ºC for 25 min and kept at 4 ºC until immunohistochemistry processing.

To perform the immunohistochemistry, the organoid sections were washed three times with PBS and blocked and permeabilized with 0.1% Triton™ X-100 (Sigma) and 10% normal goat serum (NGS) prepared in PBS for 2 h at room temperature. Then, sections were incubated overnight at 4ºC with the primary antibodies (Table 1) prepared in 10% NGS/PBS. Sections were further washed three times with PBS and incubated with the Alexa Fluor conjugated secondary antibodies, Alexa Fluor-594 anti-mouse, Alexa Fluor-488 anti-rabbit, Alexa Fluor-647 anti-guinea pig, and Alexa Fluor-647 anti-chicken (1:200, ThermoFisher), prepared in 2% NGS in PBS, for 2 h at room temperature. For synapse staining, the antibodies dilutions were centrifuged at 13,000 rpm for 10 min at 4 ºC to remove antibody aggregates, prior to incubation, and the secondary antibody incubation was performed for 45 min at 37 ºC. Afterward, sections were washed three times with PBS and incubated with the nuclear DNA staining reagent DAPI (1:5000) for 10 min. Finally, sections were washed three times with PBS, dried, and mounted with Mowiol reagent (Sigma). Widefield fluorescence images were acquired with an EC Plan-Apochromat 10×/0.3NA and Plan-Apochromat 20×/0.8NA air objectives and Plan-Apochromat 63×/1.40 Oil DIC M27 objective on a Carl Zeiss Axio imager Z2 microscope. Confocal fluorescence images were obtained using a Plan-Apochromat 63×/1.40 Oil DIC M27 objective on a LSM Zeiss microscope.

**Table 1:** Antibodies.

Antibody	Dilution	Manufacturer	Reference	Specie
ATOH1	1:500	Protein Tech	21215-1-AP	Rabbit
Calbindin	1:1000	Millipore	AB1778	Rabbit
GFAP	1:400	Dako	Z0334	Rabbit
MAP2	1:125	Sigma-Aldrich	M4403	Mouse
ATXN3	1:1000	Millipore	MAB5360	Mouse
PSD95	1:1000	Cell Signaling	3450	Rabbit
VGlut1	1:1000	Millipore	AB5905	Guinea-pig
VGAT	1:500	Synaptic Systems	131004	Guinea-pig
Gephyrin	1:500	Synaptic Systems	147011	Mouse
NeuroD1	1:300	Abcam	ab60704	Mouse
Msi1	1:500	Abcam	ab21628	Rabbit
Nestin	1:250	R&D systems	MAB1259	Mouse
Pax6	1:100	Thermofisher	42-6600	Rabbit
PCP4 (C-15)	1:100	Santa Cruz	sc-74816	Rabbit
S100Β	1:500	Abcam	ab52642	Rabbit
TBR1	1:500	Abcam	ab31940	Rabbit
β3-Tubulin	1:500	Invitrogen	32-2600	Mouse

### Ataxin-3 aggregation quantification

Brain organoid sections were labeled for ataxin-3 through immunohistochemistry, as previously described. Six to ten pictures of each organoid were acquired with Plan-Apochromat 63x/1.40 Oil DIC M27 objective and ApoTome.2 on a Carl Zeiss Axio imager Z2 microscope. Using the Icy bioimage informatics platform^19^, pictures were quantified for ataxin-3-positive spots/aggregates present in cell nucleus (ROI created from DAPI channel). The settings regarding ataxin-3-positive spots object size and sensitivity were kept constant in all quantified pictures. Finally, the number of ataxin-3-positive aggregates and DAPI area (mm^2^) were automatically determined for each picture by the software and the number of ataxin-3-positive aggregates was normalized for the DAPI area in each picture.

### Inhibitory and excitatory synapses quantification

Brain organoid sections were labeled for Gephyrin- and VGAT-positive puncta for inhibitory synapses determination and labeled for PSD95- and VGlut1-positive puncta for excitatory synapses through immunohistochemistry, as previously described. Observation and imaging of synaptic puncta were performed with a Plan-Apochromat 63×/1.40 Oil DIC M27 objective on an LSM Zeiss confocal microscope. Image analysis was performed using Fiji software. Instances of colocalization of pre- and postsynaptic markers were quantified as synapses. Values were normalized per organoid volume to the mean value of the control.

### Ventricular-like zones and organoids size quantification

Sections from organoids collected at different time points (day 25 and 40) were washed three times with PBS before incubation with the nuclear DNA staining reagent DAPI for 10 min. After, the sections were washed three times with PBS, air dried, and mounted with Mowiol reagent (Sigma). Widefield fluorescence images were acquired with an EC Plan-Apochromat 20×/0.8NA air objective. A tile image was acquired and stitched together to reconstitute the entire organoid. The DAPI channel was used to manually count the number of ventricule-like structures. Using the same channel, the image of the whole organoid was segmented and the magic wand tool was applied to the resulting mask to measure the total area of each section. This measurement was then used to normalize the number of the ventricular-like zones. To quantify the central ventricular-like zones, the initial area defined by the wand tool was scaled to produce a section of 25% of the initial area, which was centralized relatively to the original position. The ventricles located inside this region were then manually counted. Image analysis was performed using Fiji software.

### RT-qPCR

IPSC (day 0) and organoids collected at different time points (day 25 and 40) were washed three times with PBS and kept at −80 ºC until processing. RNA was extracted using NucleoSpin RNA kit (Macherey–Nagel) according to manufacturer’s instructions. The RNA purity and concentration were measured with NanoDrop™ 2000 (Thermo Scientific) and 1000 ng of total RNA was used to synthesize cDNA with iScript™ cDNA Synthesis Kit (Bio-Rad), according to manufacturer’s instructions. Quantitative real time PCR (qPCR) was performed with SsoAdvanced™ SYBR Green Supermix Kit (Bio-Rad) using cDNA obtained in the reverse transcription reaction diluted ten times with DNase free water (Sigma). Quantitative PCR was performed as follows: a single cycle of 95 ºC for 30 s, followed by 45 cycles of two steps: first step of 5 s at 95 ºC and a second step of 15 s at temperature depending on each primer set (Table 2). The threshold cycle (CT) for each gene was generated automatically by the StepOne™ Software (Applied Biosystems). In each experiment, and for all genes, a standard curve was performed and quantitative PCR efficiency was determined by the software. Additionally, no template and no reverse transcriptase negative controls were included. The relative mRNA quantification with respect to control samples was determined by the Pfaffl method, taking into consideration the different amplification efficiencies of all genes.

**Table 2:** Primers used for RT-qPCR.

Gene	Manufacturer	Reference/sequence	T (°C)
NANOG	Sigma	KiCqStart pre-designed primers	58
PAX6	Sigma	KiCqStart pre-designed primers	58
NEUROD1	Sigma	KiCqStart pre-designed primers	58
TUBB3	Invitrogen	Fw: GGCCAAGGGTCACTACACG Rev: GCAGTCGCAGTTTTCACACTC	58
MAP2	Invitrogen	Fw: CGAAGCGCCAATGGATTCC Rev: TGAACTATCCTTGCAGACACCT	57
GFAP	Invitrogen	Fw: AGGTCCATGTGGAGCTTGAC Rev: GCCATTGCCTCATACTGCGT	58
CALB1	Sigma	KiCqStart pre-designed primers	57
PCP4	Sigma	KiCqStart pre-designed primers	59
HPRT	Qiagen	Hs_HPRT1_1_SG QuantiTect primer assay, QT00059066	60
SYP	Sigma	KiCqStart pre-designed primers	58
MSI1	Sigma	KiCqStart pre-designed primers	60
NOTCH1	Sigma	KiCqStart pre-designed primers	60
BDNF	Sigma	KiCqStart pre-designed primers	58
GPHN	Sigma	KiCqStart pre-designed primers	58
ATOH1	Sigma	KiCqStart pre-designed primers	58
SLC17A7	Sigma	KiCqStart pre-designed primers	56.8
SLC32A1	Sigma	KiCqStart pre-designed primers	58
SQSTM1	Sigma	KiCqStart pre-designed primers	60
MAP1LC3B	Sigma	KiCqStart pre-designed primers	58
NEFL	Sigma	KiCqStart pre-designed primers	57

T (°C): annealing temperature.

### Single-cell calcium imaging

The presence of neurons firing upon stimulation with potassium (50 mM KCl), an indication of functional neurons, was assessed in the established organoids through single-cell calcium imaging. Organoids were collected at day 35, washed twice in 0.1% BSA/Krebs buffer (142 mM NaCl, 1 mM KCl, 1 mM MgCl_2_, 10 mM Glucose, 10 mM Hepes, 10 mM NaHCO_3_, 1 mM CaCl_2_, pH 7.4) and incubated with 5 μM Fluo-4/AM (Invitrogen) and 0.02% pluronic acid in 0.1% BSA/Krebs buffer for 1 h, under orbital agitation at 37 ºC and 5% CO_2_. Then, organoids were transferred to 0.1% BSA/Krebs buffer and kept at 37 ºC for observation. The basal fluorescence levels were measured for 4 min, and then the 50 mM KCl (in 0.1% BSA/Krebs buffer) stimulus was added for 2 min to access alterations in basal fluorescence. For the voltage-gated specific neuronal firing evaluation, 1 μM of Tetrodotoxin (TTX)^20^ was used to block voltage-gated sodium channels and 100 μM Cadmium chloride (CdCl_2_) was used to block voltage-gated calcium channels^21^. Upon the blocker administration (~ 4 min later) the potassium stimulus was added to the organoids in 0.1% BSA/Krebs buffer and fluorescence was recorded for 2 min. The assay was performed in a Carl Zeiss Cell Observer Spinning Disk microscope (EM-CCD Evolve Delta camera) using a Plan-Apochromat 20×/0.8NA air objective, recording fluorescence at 488 nm for the whole experiment. Fluo-4/AM is a calcium indicator, increasing its fluorescence excitation at 488 nm upon Ca^2+^ binding, thus increasing the fluorescence signal level upon neuronal depolarization. Image analysis was performed in Fiji software. Cell bodies were drawn and the shift in signal intensity was measured. Cells were considered to respond to potassium stimulus when a 15% increase in the fluorescence signal was registered.

### Statistical analysis

All data are presented as mean ± SEM. Graphs and statistical analysis were performed in Graph Pad Prism 6 software. Statistical significance was assessed by unpaired t-test and One-way ANOVA and significant thresholds were set at p < 0.05, p < 0.01, p < 0.001 and p < 0.0001.

## Results

### Control and MJD iPSC-derived whole-brain human organoids originate brain cell lineages and patterning recapitulating aspects of human brain organogenesis

Whole-brain organoids were obtained from Control and MJD iPSC with a commercially available kit, as illustrated in Fig. 1a. To evaluate whether the established brain organoids recapitulate and model hallmarks of human brain organogenesis, the mRNA levels of pluripotency (*NANOG*), neuroectoderm (*PAX6*), neurons (*TUBB3* and *MAP2*), and astroglia (*GFAP*) (Fig. 1b–f) markers were evaluated throughout organoids induction and maturation (days 0, 25 and 40). As expected, for both Control and MJD organoids (ORG) the *NANOG* mRNA levels were decreased as organoids induction and maturation progressed. For Control ORG at day 25, *NANOG* mRNA levels were found 83.33 ± 0.004-fold decreased and, at day 40, the levels were 333.33 ± 0.001-fold decreased when compared with day 0. For MJD ORG, at day 25 NANOG mRNA levels were 500 ± 0.002-times reduced, and at day 40 were decreased in 333.33 ± 0.002-times (Fig. 1b). The reduction of the *NANOG* mRNA levels was complemented with the increase in the mRNA levels of the neuroectoderm marker *PAX6*. At day 25, Control and MJD organoids presented a significant 721.48 ± 269.83 and 20,039.54 ± 1642.87-fold increase, respectively, that was maintained at day 40 (Fig. 1c), indicating that there is a shift from pluripotency to multipotency state, more specifically to the neuroectoderm germinal layer, as expected in neural induction phase (Fig. 1a). On day 40, there was a non-significant reduction in *PAX6* mRNA levels and augmentation of markers related to neuronal differentiation, such as *MAP2*, (Fig. 1e) and glial cells differentiation, like *GFAP* (Fig. 1f).

Additionally, the mRNA levels of the immature neuronal marker *TUBB3* (Fig. 1d) were found increased by 23.20 ± 4.038-fold at day 25 for MJD ORG; whereas the mature neuronal marker *MAP2* presented higher mRNA levels at day 40, which showed a 108.82 ± 20.27-fold increase in MJD ORG. Finally, recapitulating brain organogenesis where glial cells emerge after neurons, the mRNA levels of the glial marker *GFAP* were found significantly elevated only at day 40, a later time-point when compared to neuronal markers, such as *TUBB3* that was found raised at day 25.

The neuroectoderm commitment and neuronal and glia presence in the established organoids was further confirmed by immunohistochemistry through Pax6, Nestin, MAP2, GFAP, and S100B protein expression (Fig. 1g–u). The expression of the neuroepithelial stem cell protein Nestin specifically distinguishes neural progenitors from more differentiated cells in the neural tube^22^ and the Pax-6 transcription factor coordinates neurogenesis, namely cell differentiation and proliferation, and it is a marker for the outer radial glial cells (oRGs)^23,24^. Thus, the presence of Nestin and Pax-6 at 25 (Fig. 1g,h and Supplementary Fig. S1) and 40/50 days (Fig. 1i,j and Supplementary Fig. S1) indicates that both Control and MJD ORG were committed to the neuroectoderm lineage. This was corroborated by the presence of ventricular-like zones (VZ) (white circles) containing Pax6- and Nestin-positive cells, which is an indication of the successful induction of the pluripotent stem cells into the neuroectoderm lineage. The neuronal marker MAP2 was detected surrounding the VZ at days 25 and 40 for both CNT and MJD ORG. Regarding the glial marker GFAP, few GFAP-positive cells were found at day 25, whereas at day 40, namely at the organoids peripheral edge, were abundantly detected (Fig. 1q–s and Supplementary Fig. S2). This result corroborates the MAP2 and GFAP mRNA levels data, indicating that neurons developed before glia. A second marker of glial cells, S100B, considered a marker of more mature glial cells^25^, was also detected in the established organoids (Fig. 1t,u). Thus, as previously described by other authors^23^, the obtained organoids present ventricular-like zones with neuroepithelial stem cells positive for Pax6, which are surrounded by intermediate progenitors (Nestin-positive cells), neurons (MAP2-positive), and glial cells (GFAP- and S100B-positive). The ventricular zone-like structures were present in whole-brain organoids and were found more abundant at the periphery (Fig. 1s). Finally, to demonstrate the ability to obtain functional neurons in the organoids, single-cell calcium imaging^26^ was performed (Fig. 1v–x). Data showed that upon administration of potassium, which depolarizes functional neurons, 100.0% of the CNT and 98.47 ± 1.25% of MJD organoids’ analyzed cells responded to potassium (Fig. 1x), demonstrating the presence of firing neurons. Moreover, to demonstrate the voltage-gated specific neuronal firing, a voltage-gated sodium channel blocker (Tetrodotoxin—TTX)^20^ and a voltage-gated calcium channel blocker (Cadmium chloride—CdCl_2_)^21^ were used. Data indicate no significant reduction in the percentage of cells responding to potassium stimulus upon sodium channels blockage. Whereas, upon blockage of calcium channels a significant reduction in the percentage of cells increasing the calcium intracellular concentration upon potassium stimulation was detected, with only 1.81 ± 0.91% of the cells responding to the stimulus (Fig. 1x), demonstrating the calcium voltage-gated neuronal firing.

### Whole-brain organoids present markers of neural progenitors proliferation and differentiation, neurotrophic factors, and synapses

**Figure 2:**
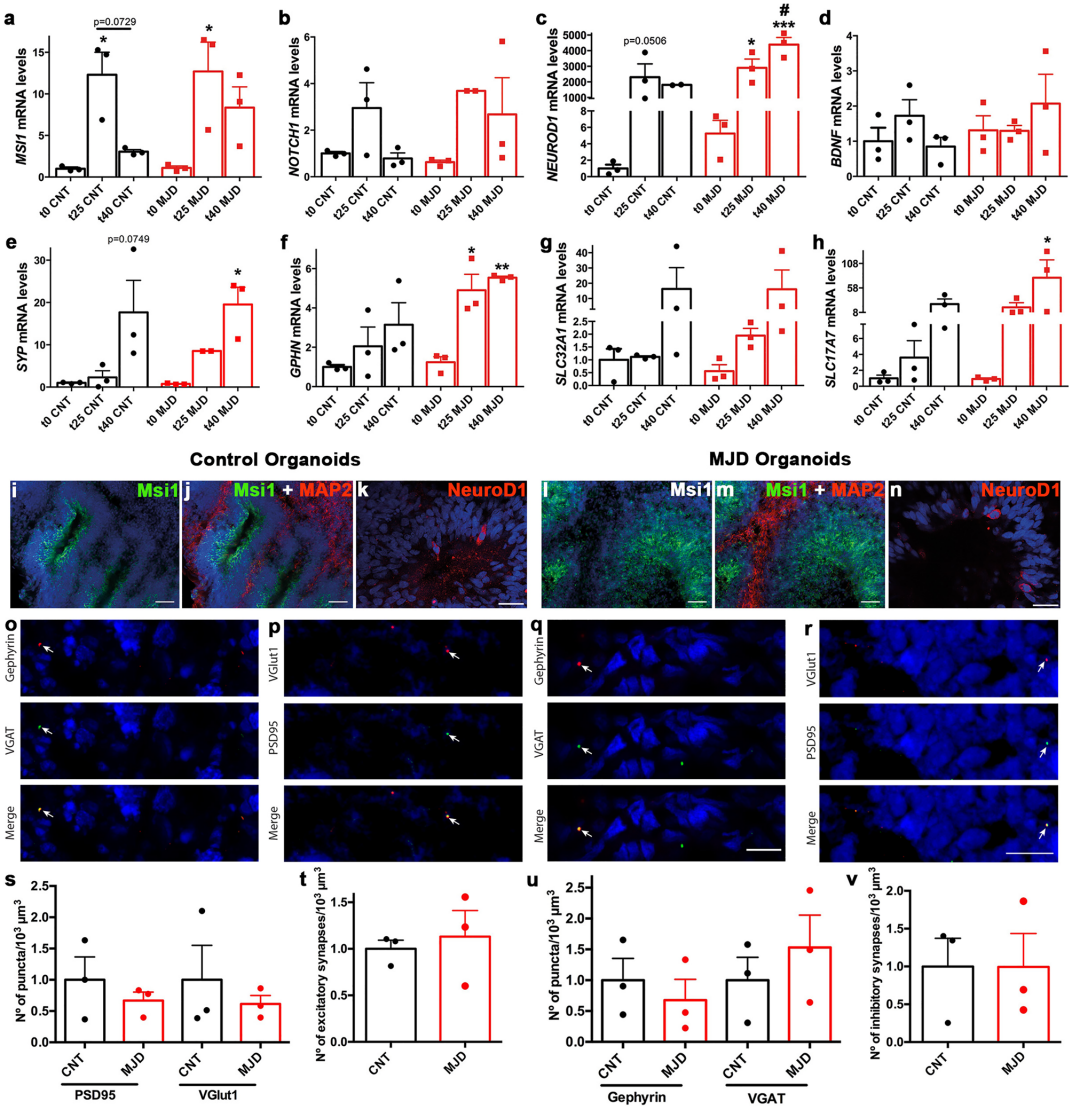
Control and MJD brain organoids present markers of neural progenitors proliferation, neuronal differentiation, and synaptic function. **(a–h)** Control and MJD organoids at different time points (day 0, 25, and 40; t0, t25, and t40 respectively) evaluated for neural progenitors proliferation markers **(a)** *MSI1* and **(b)** *NOTCH1*, neuronal differentiation marker **(c)** *NEUROD1*, brain-derived neurotrophic factor **(d)** *BDNF*, and the synaptic markers **(e)** Synaptophysin (*SYP*), **(f)** Gephyrin (*GPHN*), **(g)** GABA vesicular transporter (VGAT, *SLC32A1*), and **(h)** vesicular glutamate transporter 1 (VGlut1, *SLC17A7*) mRNA levels, through RT-qPCR and normalized for t0 CNT. Data indicated an increase of neural progenitors proliferation and neuronal differentiation at day 25 and of synaptic markers at day 40. Immunohistochemistry evaluation of CNT and MJD organoids with 40/50 days for **(i,j,l,m)** Msi1 and **(k,n)** NeuroD1, showing the presence of these markers mainly in the ventricular-like zones, whereas **(j,m)** MAP2 is mainly surrounding these zones. Immunohistochemistry evaluation of **(o,q)** inhibitory synapses given by the confocal microscopy colocalization between Gephyrin and VGAT positive puncta and **(p,r)** excitatory synapses indicated by the confocal microscopy colocalization between VGlut1 and PSD95 (postsynaptic density protein 95) positive puncta presence in Control and MJD organoids, demonstrating the presence of inhibitory and excitatory synapses in Control and MJD organoids; white arrows indicate the synapses. Number of **(s)** PSD95 and VGlut1 puncta, **(t)** excitatory synapses, **(u)** Gephyrin and VGAT puncta, and **(v)** inhibitory synapses per organoid volume (1000 μm^3^) normalized for the control organoids. DAPI: blue, representative images of 3 independent experiments, scale bars: 50 μm, (**o–r**) 10 μm. Data are expressed as mean ± SEM (n: 3 independent experiments), *p < 0.05, **p < 0.01, ***p < 0.001 (comparison established with the correspondent t0 or between the conditions indicated by the lines), and ^#^p < 0.05 (comparison established between CNT and MJD ORG at the same time point), One-way ANOVA followed by Tukey posttest and (**s–v**) unpaired t test with Welch's correction.

Other important markers related with brain organogenesis were evaluated, such as the neural progenitors proliferation markers Musashi 1 (MSI1) and NOTCH1^27,28^, the neuronal differentiation marker NEUROD1^29,30^, and the brain-derived neurotrophic factor (BDNF) that is involved in neuronal maturation, survival, and performance^31^. *MSI1* mRNA levels were found significantly increased at day 25, for both CNT (12.30 ± 2.71-fold) and MJD (12.71 ± 3.52-fold) ORG (Fig. 2a); whereas no significant changes were detected for *NOTCH1* mRNA levels (Fig. 2b).

*NEUROD1* mRNA levels were higher at day 25 for CNT (2300.99 ± 856.79-fold) and MJD (2900.53 ± 562.54-fold) ORG (Fig. 2c). No significant changes were detected in *BDNF* mRNA levels of CNT and MJD ORG neither at day 25 nor day 40 (Fig. 2d).

The presence of both inhibitory and excitatory synapses is an evidence of synaptic assembly and an indication of the ability of the established organoids to originate potentially firing neural networks^32^. Thus, we assessed the mRNA levels of synapse-related markers, such as Synaptophysin (*SYP*), a presynaptic vesicle membrane protein described to be involved in synaptic vesicle formation and exocytosis^33^ (Fig. 2e), Gephyrin (*GPHN*), which is an inhibitory postsynaptic protein (Fig. 2f), Vesicular GABA Transporter (*SLC32A1*), an inhibitory presynaptic protein (Fig. 2g), and Vesicular Glutamate Transporter 1 (*SLC7A17*), an excitatory postsynaptic protein (Fig. 2h). At day 40, a 19.53 ± 4.08-fold increase in the *SYP* mRNA levels was detected in MJD ORG. *GPHN* mRNA levels were found significantly increased in MJD ORG, for both day 25 (4.90 ± 0.81-fold) and 40 (5.54 ± 0.076-fold), as compared with day 0. Finally, for both CNT and MJD ORG a tendency for *SLC32A1* and *SLC7A17* mRNA levels increase, at day 40, was observed. Nevertheless, only *SLC7A17* mRNA levels in MJD ORG were found significantly increased with a 79.00 ± 36.45-fold augmentation.

The presence of markers of neural progenitors proliferation and neuronal differentiation in the established organoids was further confirmed by immunofluorescence staining of Msi1 and NeuroD1 proteins (Fig. 2i–n). Data demonstrated the presence of Msi1- and NeuroD1-positive cells in the ventricular-like zones in both CNT and MJD organoids at day 40, indicating the presence of cells differentiating into neurons.

The presence of inhibitory (colocalization of VGAT- and gephrynin-positive puncta) and excitatory (colocalization of VGlut1- and PSD95-positive puncta) synapses was also assessed by confocal microscopy in control and MJD organoids at day 40. Data indicated that the obtained organoids were able to establish both inhibitory (Fig. 2o,q) and excitatory (Fig. 2p,r) synapses. The number of PSD95 and VGlut1 puncta (Fig. 2s) and respective excitatory synapses number (Fig. 2t) in CNT and MJD organoids exhibit no significant difference. Similarly, no significant difference was detected in the number of Gephyrin and VGAT puncta (Fig. 2u) and respective inhibitory synapses number (Fig. 2v) between CNT and MJD organoids. Altogether, data have shown that the organoids exhibited active neurogenesis, with signs of neuronal differentiation enabling the formation of inhibitory and excitatory synapses. Moreover, no difference in synapses number between CNT and MJD organoids was detected at day 40.

### Whole-brain organoids present cerebellar neuronal progenitors and neurons

**Figure 3:**
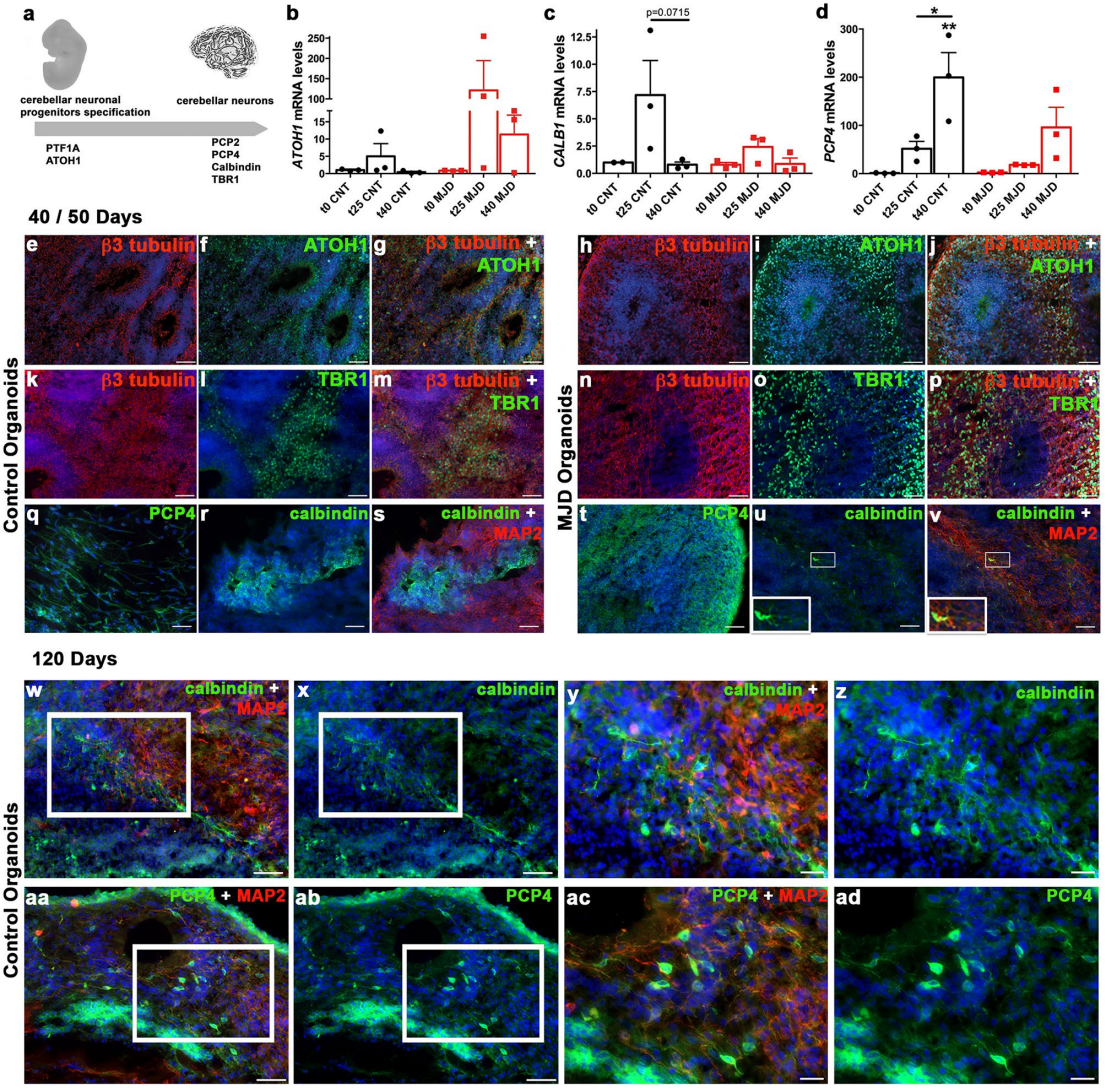
Control and MJD brain organoids present markers of cerebellar neurons. **(a)** Schematic representation of cerebellar neuronal progenitors (PTF1a and ATOH1) and cerebellar neurons (PCP2, PCP4, Calbindin, and TBR1) markers expression in cerebellum development. **(b)** *ATOH1*, **(c)** *CALB1*, and **(d)** *PCP4* mRNA levels evaluation of Control (CNT) and MJD organoids, normalized for t0 CNT, collected at different time points (day 0, 25, and 40; t0, t25, and t40 respectively). Data indicated a significant enhancement of *PCP4* mRNA levels at day 40. Data are expressed as mean ± SEM (n: 3 independent experiments), *p < 0.05 and **p < 0.01 (comparison established with the correspondent t0 or between the conditions indicated by the lines), One-way ANOVA followed by Tukey posttest. Immunohistochemistry evaluation of Control and MJD organoids with **(e–v)** 40/50 days and **(w–ad)** 120 days for the expression of markers of neurons **(e,g,h,j,k,m,n,p)** β3 tubulin and **(s,v,w,y,aa,ac)** MAP2, cerebellar neuronal progenitors **(f,g,i,j)** ATOH1, and of cerebellar neurons **(l,m,o,p)** TBR1, **(q,t,aa–ad)** PCP4, and **(r,s,u,v,w–z)** Calbindin; **(u–v)** lower insert: cell presenting Calbindin and MAP2 colocalization. (**y)** magnification of the (**w**) image indicated in the white square; (**z)** magnification of the (**x**) image indicated in the white square; (**ac)** magnification of the (**aa**) image indicated in the white square; and (**ad)** magnification of the ab image indicated in the white square. Data indicated the presence of cells positive for cerebellar neuronal progenitors and cerebellar neurons in control and MJD organoids. DAPI: blue, representative images of 3 (**e–v**) and 2 (**w–ad**) independent experiments, scale bars: 50 μm and (**y,z,ac,ad**) 20 μm.

The presence of cerebellar neurons in the established organoids is essential to model cerebellar diseases. The protocol used in the present work has been extensively used to model the brain^13,34,35^. However, cerebellar modeling and, consequently, evaluation of cerebellar neurons presence have not been fittingly investigated in the organoids obtained with this protocol. Thus, in the present work we evaluated the presence of both cerebellar neuronal progenitors (ATOH1) and cerebellar neurons (PCP4, Calbindin, and TBR1), as illustrated in Fig. 3a. Atonal BHLH Transcription Factor 1 (ATOH1) is exclusively expressed in the rhombic lip and is related to the origin of glutamatergic neurons in the developing cerebellum^36^. Calbindin (CALB1) and Purkinje Cell Protein-4 (PCP4) are markers of Purkinje cells, GABAergic neurons with central role in cerebellar neuronal communication^37^. Finally, T-Box Brain Transcription Factor 1 (TBR1) is a marker used for deep cerebellar nuclei neurons identification^38^. To evaluate the presence of these cell populations in CNT and MJD organoids with 25 and 40 days, we quantified *ATOH1*, *CALB1*, and *PCP4* mRNA levels (Fig. 3b–d). The results revealed that while *PCP4* mRNA levels were increased at day 40 in the CNT ORG by 199.58 ± 4.08-fold change (Fig. 3d), no significant changes were observed for the *ATOH1* and *CALB1* mRNA levels (Fig. 3b,c). The presence of ATOH1-, TBR1-, Calbindin-, and PCP4-positive cell populations in the established brain organoids with 40–50 days was evaluated through immunohistochemistry. Data indicated that ATOH1, TBR1, PCP4, and Calbindin are present both in CNT and MJD organoids, indicating the presence of cells positive for cerebellar neuronal progenitors (Fig. 3e–j), deep cerebellar neurons (Fig. 3k–p), and Purkinje cells (Fig. 3q–v) markers; even though the latter, mainly calbindin-positive cells, are less abundant. Purkinje cells are the largest neurons in the human cerebellum and exhibit a characteristic morphology, namely, Purkinje cells extend one primary dendrite from the soma, which arborizes into a highly branched structure^39^. To evaluate the presence of Purkinje cell dendritic arbors, organoids with 120 days were evaluated for Calbindin- (Fig. 3w–z) and PCP4-positive dendritic arborization (Fig. 3aa–ad). Data demonstrated that at day 120 there is a clear enhancement of Calbindin-positive cells compared with day 40. Nevertheless, the neuronal projections of both Calbindin- and PCP4-positive cells did not present the characteristic morphology of Purkinje cells, which might indicate that longer maturation is required for the organoids present Purkinje cell dendritic arbors.

### MJD-associated neuropathology hallmarks in MJD whole-brain organoids

**Figure 4:**
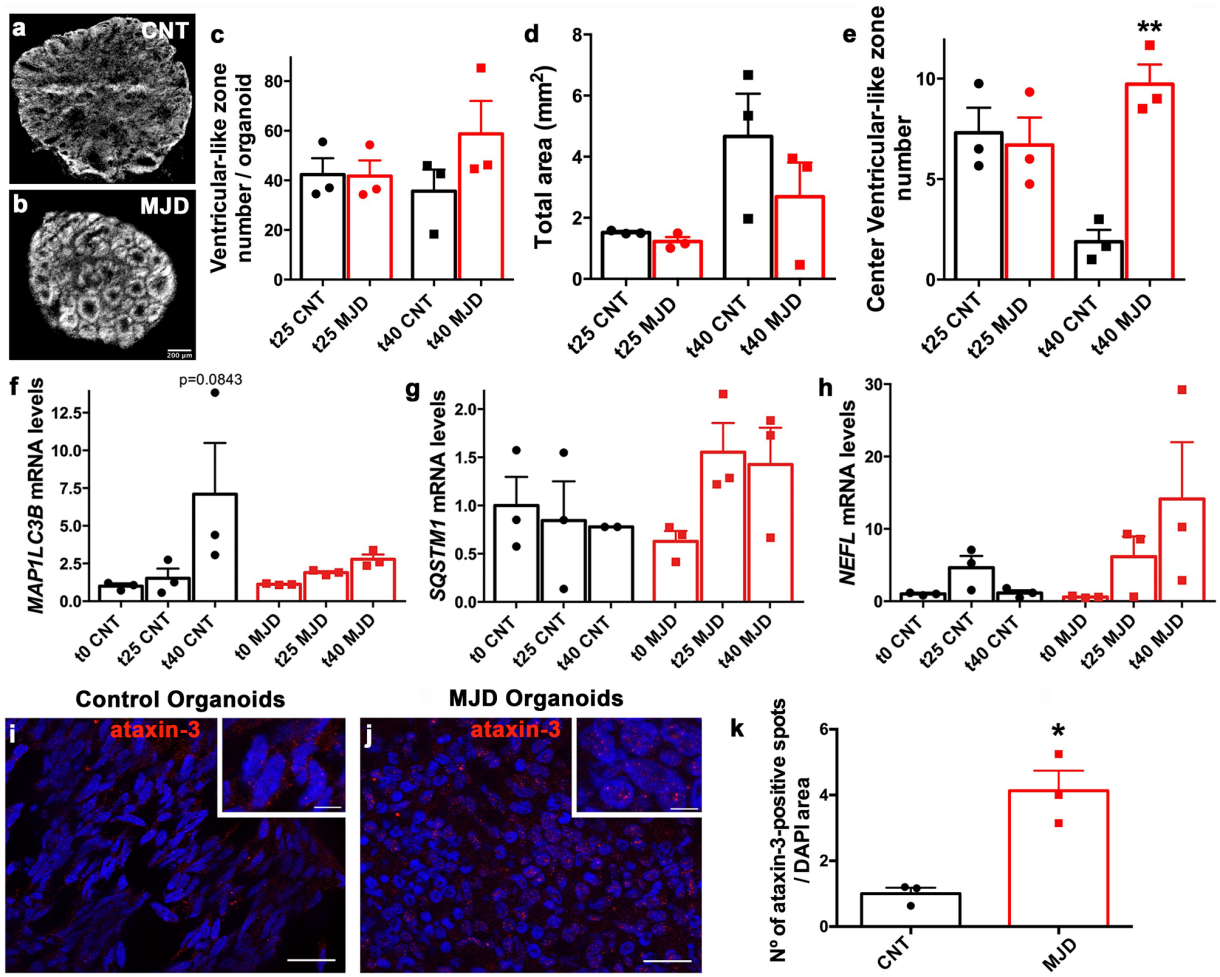
Neuropathology hallmarks in MJD brain organoids evaluation. **(a–e)** Control and MJD brain organoids derived from control and MJD iPSC, respectively, present ventricular-like zones assessed by nuclear DNA labeling with DAPI. **(a,b)** Representative images illustrating brain organoids´ ventricular-like zones in **(a)** control and **(b)** MJD organoids; scale bar: 200 μm. Quantification of **(c)** ventricular-like zones number *per* organoid, **(d)** organoids area, and **(e)** central ventricular-like zones number (in 25% of the center of the organoids) of control and MJD organoids with 25 and 40 days, t25 and t40 respectively. Autophagy markers **(f)** *MAP1LC3B* (LC3B) and **(g)** *SQSTM1* (P62) and neurodegeneration marker **(h)** *NEFL* (NfL) mRNA levels evaluation. Control and MJD organoids collected at different time points (day 0, 25, and 40; t0, t25, and t40 respectively) were evaluated for the mRNA levels through RT-qPCR, and normalized for t0 CNT. MJD organoids presented a tendency for lower *MAP1LC3B* and higher *SQSTM1* mRNA levels at day 40, as compared with control organoids. Data are expressed as mean ± SEM (n: 3 independent experiments), **p < 0.01 (**c–e**: comparison established between CNT and MJD at the same time point, **f–h**: comparison established with the correspondent t0), One-way ANOVA followed by Tukey posttest. **(i,j)** Representative immunofluorescence pictures of control (CNT) and MJD brain organoids with 40 days stained for human ataxin-3, (red) showing ataxin-3-positive protein spots/aggregates. Upper inserts: higher magnification image showing ataxin-3-positive spots/aggregates (red) in DAPI-positive cell nucleus (blue). Representative images of 3 independent experiments, scale bars: 20 μm and 5 μm in upper inserts. (**k)** Quantification of ataxin-3-positive spots in cell nucleus (DAPI) of control and MJD brain organoids at day 40 demonstrated significantly higher number of ataxin-3-positive spots in MJD organoids, as compared with control organoids. Data are expressed as mean ± SEM (n: 3 independent experiments), *p < 0.05, Unpaired t test with Welch's correction.

Immunohistochemistry of human brain organoids revealed the presence of ventricular-like zones, which are associated with primates’ brain development^10,13,14^. Morphologically, organoids maturation can be estimated by the number of ventricular-like zones and their regionalization, for instance, a higher number of ventricular-like zones is an indication of less mature organoids^23^. Therefore, in order to understand whether the MJD organoids present differences in maturation, ventricular-like zones number and organoids size were assessed (Fig. 4a–e). Although no significant differences were found in the ventricular-like zones number per area (Fig. 4c) or in the organoids size (Fig. 4d), a significant higher number of ventricular-like zones in the central area of the MJD organoids was detected at day 40, when compared with CNT ORG (Fig. 4a,b,e), an indication of decreased maturation of MJD organoids.

Furthermore, the presence of MJD-neuropathology hallmarks in MJD organoids was evaluated (Fig. 4f–k). To this end, the levels of autophagy markers MAP1LC3B (LC3B) and SQSTM1 (p62*),* neurofilament light chain protein (NfL), a neurodegeneration biomarker associated with axonal damage and structural brain changes^40^, and the presence of mutant ataxin-3 inclusions, were assessed. As showed in Fig. 4, no significant differences were observed between CNT and MJD organoids for the mRNA levels of the autophagy-related genes *MAP1LC3B* (Fig. 4f) and *SQSTM1* (Fig. 4g) and neither for Neurofilament Light Chain *(NEFL)* (Fig. 4h). Although, as expected, MJD organoids presented a trend for lower *MAP1LC3B* and higher *SQSTM1* mRNA levels at day 40, as compared with control organoids.

Finally, the presence of mutant ataxin-3-driven protein aggregation was evaluated in control and MJD brain organoids with 40 days (Fig. 4i-k). Given the low maturation of brain organoids with 40 days, no significant presence of mutant ataxin-3 protein nuclear inclusions, spherical intranuclear structures varying in size from 0.5 to **∼**6 μm in diameter^5^, was detected in the MJD organoids. Nevertheless, it has been reported that ataxin-3 microaggregates are neurotoxic and might represent an early step of MJD pathology^41,42^. Accordingly, the quantification of the ataxin-3-positive spots/aggregates was performed. Data revealed that MJD brain organoids present 4.13 times increased levels of ataxin-3-positive spots as compared with Control organoids. This observation indicates that although the nuclear mutant ataxin-3 inclusions characteristic of the MJD pathology are not yet significantly present, the protein immunoreactive spots/aggregates are increased.

## Discussion

The available cellular models have important limitations in human brain diseases modeling^10^, while animal models, although more complete still present important interspecies differences, resulting in translational barriers^11^. Accordingly, to overcome some of the limitations of the currently used models for human brain research, 3D human brain organoids have been developed to study the human brain development and to test new therapeutic approaches for brain diseases^43,44^. Different protocols for brain organoid generation have been implemented describing 3D models mimicking the human brain in their molecular and cellular composition^45^. The protocol used in the present work is easy to implement^13^, and enables the generation of human whole-brain organoids from pluripotent stem cells. It was first established in 2013 to study microcephaly^13^, and has also been used to other brain modeling purposes^34,46^.

The present work evaluated whether the established protocol for whole-brain organoids generation is suitable for modeling cerebellar diseases, such as MJD. The data obtained from the mRNA levels evaluation of organoids at different time points (day 0, 25, and 40) performed by RT-qPCR indicate, as expected, a reduction of the pluripotency maker *NANOG* expression over time, in both MJD and CNT organoids, which resembles development and has been previously described with this protocol^13^. In accordance, neuroectoderm *PAX6* marker increase at day 25 was observed for both Control and MJD organoids. Pax6 upregulation was expected as the pluripotent marker started to decrease, indicating that the pluripotent stem cells begin to be committed into the neuroectoderm cell lineage. In the first stages of brain development it is also expected that neuronal differentiation marker levels increase. Thus, as the organoids differentiation and maturation progress an elevation in *TUBB3* and *MAP2* neuronal markers is anticipated, which was indeed observed from day 0 to day 25 and maintained at day 40.

Regarding the glial marker GFAP, an increment in its levels was observed only at day 40, later than the neuronal markers. These results are in accordance with embryonic neurogenesis and gliogenesis, given that neuronal progenitors proliferation and migration precede gliogenesis (astrogliogenesis and oligodendrogenesis)^47^.

Immunohistochemistry labeling showing the presence of Pax6-positive cells in the VZ and Nestin-positive cells surrounding the VZ are a clear indication of the neuroectoderm induction in these organoids. In fact, the human and macaque primary cortex present a characteristic cellular organization of radial glia and progenitor cells in the ocular and subventricular zones^23,48^. In agreement with other reports, immunohistochemistry of human brain organoids reveals the presence of ventricular zone-like structures presenting neuroepithelial stem cells forming the inner VZ, as well as the presence of neurons and glial cells in the periphery of these structures^23^. Hence, the data indicate that the obtained organoids recapitulate some features of human brain development, presenting VZ with different brain cell populations from neural progenitors (Nestin-positive), to glia (GFAP- and S100B-positive) and neurons (β3 Tubulin- and MAP2-positive). Moreover, single-cell calcium imaging demonstrated the presence of firing neurons and of calcium voltage-gated neuronal firing, which is an indication of neuronal activity in the established brain organoids.

There are other important cellular mechanisms required for the proper development of an accurate human brain model, such as the presence of neurogenesis^49^, neurotrophic factors, and functional synapses. Neurogenesis was found represented in the organoids (day 25 and 40) by the upregulation of neuronal progenitors proliferation markers, such as MSI1 and NOTCH1^27,28,50^, and the neuronal differentiation marker NEUROD1^29,30^. The neurotrophic factor BDNF is enrolled in neurogenesis, supporting neuronal differentiation, maturation, and survival, and regulating both excitatory and inhibitory synaptic transmission^31^. Nonetheless, a significant augmentation of *BDNF* mRNA levels was not detected in the established organoids. As BDNF production and release is performed by neurons^51^, we speculate that this might be explained by the reduced maturation of the organoids. Nevertheless, some degree of functional maturation was reached since *SYP* mRNA levels, associated with synaptic vesicle formation, were significantly increased at later stages (day 40), suggesting synaptic formation. Additionally, both excitatory (PSD95- and VGlut1-positive puncta) and inhibitory (VGAT- and Gephyrin-positive puncta) synapses were detected, though in low numbers, for both control and MJD organoids, which is also an indication of partial neuronal and organoid maturation. Altogether, these data demonstrated the presence of important brain developmental cellular mechanisms: the active neurogenesis and synapses establishment.

Regarding the cerebellar formation, cerebellar neurons are generated from two zones, the VZ and the rhombic lip (RL). The VZ originates all GABAergic inhibitory neurons and the RL originates all glutamatergic excitatory neurons^36^. ATOH1 protein is exclusively expressed in the RL in the developing cerebellum regulating glutamatergic neurons neurogenesis^36^. Two important cerebellar neuronal populations, are the deep cerebellar nuclei neurons, significantly affected in MJD patients^3^, and the Purkinje cells (PC), also affected in MJD patients^52,53^. While deep cerebellar nuclei (DCN) are composed by both inhibitory and excitatory neurons^54^, PC are only GABAergic inhibitory neurons with a central role regulating the whole cerebellar communication through the integration of output and input signals^37^.

The presence in our model of ATOH1, and also the deep cerebellar nuclei marker TBR1, and the PC markers PCP4 and Calbindin, is an indication that the brain organoids have the ability to originate cerebellar neurons. Thus, this protocol might be useful to the study of cerebellar neurons dysfunction in pathology and therapies development for cerebellar disorders.

To assess if the MJD ORG display signs of associated neuropathology several parameters were tested, such as organoids maturation and size, markers of autophagy impairments, and mutant ataxin-3 aggregation. Morphologically, organoids maturation is associated to the number of ventricular-like zones and their regionalization^23^. Our data indicate that MJD-derived organoids exhibited higher number of ventricular-like zones in its center, which is in accordance with other disease organoid models that present higher number of ventricular-like zones, an indication of lesser maturation^23^. Whether the reduced organoids maturation is caused, or not, by the expression of the mutant ataxin-3 is still to be confirmed, ideally by the replication of this study with organoids generated from other control and MJD iPSC cell lines, reducing the possibility that this observation is a result of the described diversity observed between different iPSC cell lines^55^, rather than the presence of mutant ataxin-3.

Autophagy impairments in MJD have been widely described and several therapeutic strategies have been described to tackle this pathology hallmark^56,57^. On the other hand, the neurofilament light chain protein (NfL), is a particularly abundant axonal cytoskeletal protein, whose increase in cerebrospinal fluid (CSF) and blood levels has been associated with axonal damage and disease-related structural brain changes^40^. Consequently, NfL has been appointed as a biomarker for neurodegenerative diseases progression, including for MJD^58,59^. The mRNA levels of *NEFL*, *MAP1LC3B,* and *SQSTM1* in the established organoids revealed no significant differences between control and MJD organoids. This observation might be a consequence of the reduced maturation presented by the organoids due to the iPSC used for organoids derivation, and the reported erasement of diseases´ phenotype by the induction of pluripotency^60,61^. Moreover, the lack of immune system and absence of vascularization in organoids^62,63^, might naturally impact the results, given the important contribution of neuroinflammation in the phenotype of neurodegenerative diseases^64^.

Ataxin-3 is involved in several cellular processes, such as deubiquitination. The presence of an expanded polyglutamine stretch results in pathological modifications in the ataxin-3 processing and subcellular distribution, culminating in the ataxin-3 aggregation and production of ataxin-3-positive nuclear inclusions in neurons that sequester several other proteins, which is an MJD hallmark^3,5^. Our data revealed no significant presence of mutant ataxin-3-positive nuclear inclusions in MJD organoids. This might be a consequence of the low maturation of the organoids at the evaluated time point (40 days). In fact, previous publications demonstrated the requirement of cytotoxic stimulus for mutant ataxin-3 inclusions to emerge in 2D cultures^18^. On the other hand, the quantification of ataxin-3-positive spots/aggregates through immunohistochemistry revealed increased ataxin-3 aggregation in MJD organoids as compared with Control organoids, suggesting that this pathological MJD hallmark is present in MJD organoids.

Altogether, our data indicate that the used protocol gave rise to organoids that are promising models with potential to be used in the study of cerebellar diseases, such as MJD. Further investigation will be required to investigate these findings in organoids derived from additional control and MJD iPSC lines and to better characterize these models at longer time points.

## Conclusions

There are several brain disorders with significant and impacting cerebellar involvement, diseases such as spinocerebellar ataxias, Alzheimer´s disease, Parkinson´s disease, and cerebellar degeneration caused by trauma, drug damage, virus infection, cancer, and genetic abnormalities. Therefore, the establishment of human brain organoids with a significant representation of the cerebellum might result in the development of more effective therapies for the treatment of these diseases.

In the present work, the MJD and control whole-brain organoids obtained exhibited cellular composition and organization recapitulating some aspects of human brain development, such as organization in ventricular-like zones and astroglia emerging after neurons. Furthermore, firing neurons, inhibitory and excitatory synapses, and active neurogenesis were detected. Additionally, the presence of markers of cerebellar neuronal progenitors (ATOH1-positive cells) and cerebellar neurons, such as deep cerebellar nuclei neurons (TBR1-positive cells) and Purkinje cells (Calbindin- and PCP4- positive cells) were detected, demonstrating cerebellar features. Regarding neuropathology, data shown that the MJD organoids present a less mature phenotype, indicated by a higher number of ventricular-like structures, and also exhibit increased ataxin-3 aggregation.

Altogether, the present work demonstrated that these organoids exhibit potential to be implemented in the study of new therapies for cerebellar diseases treatment.

## Supplementary Information

Supplementary Figures.

## Data Availability

The data are available from the corresponding authors upon reasonable request.

## References

[1] Shooshtari, S., Stoesz, B. M., Rad, P. & Khoeiniha, S. *Development of the Cerebellum from Molecular Aspects to Diseases* (ed. Marzban, H.). 423–463. (Springer, 2017).

[2] van de Warrenburg BP (2002). Spinocerebellar ataxias in the Netherlands: Prevalence and age at onset variance analysis. Neurology.

[3] Matos CA, de Almeida LP, Nobrega C (2019). Machado-Joseph disease/spinocerebellar ataxia type 3: Lessons from disease pathogenesis and clues into therapy. J. Neurochem..

[4] Coutinho P, Andrade C (1978). Autosomal dominant system degeneration in Portuguese families of the Azores Islands. A new genetic disorder involving cerebellar, pyramidal, extrapyramidal and spinal cord motor functions. Neurology.

[5] Paulson HL (1997). Intranuclear inclusions of expanded polyglutamine protein in spinocerebellar ataxia type 3. Neuron.

[6] Lopes TM (2013). Widespread neuronal damage and cognitive dysfunction in spinocerebellar ataxia type 3. J. Neurol..

[7] Matos CA (2016). Ataxin-3 phosphorylation decreases neuronal defects in spinocerebellar ataxia type 3 models. J. Cell Biol..

[8] Takahashi K, Yamanaka S (2006). Induction of pluripotent stem cells from mouse embryonic and adult fibroblast cultures by defined factors. Cell.

[9] Henriques D, Moreira R, Schwamborn J, Pereira de Almeida L, Mendonca LS (2019). Successes and hurdles in stem cells application and production for brain transplantation. Front. Neurosci..

[10] Li M, IzpisuaBelmonte JC (2019). Organoids—Preclinical models of human disease. N. Engl. J. Med..

[11] Dawson TM, Golde TE, Lagier-Tourenne C (2018). Animal models of neurodegenerative diseases. Nat. Neurosci..

[12] Mendonca LS (2018). Stem cell-based therapies for polyglutamine diseases. Adv. Exp. Med. Biol..

[13] Lancaster MA (2013). Cerebral organoids model human brain development and microcephaly. Nature.

[14] Lancaster MA, Knoblich JA (2014). Organogenesis in a dish: Modeling development and disease using organoid technologies. Science.

[15] Muguruma K, Nishiyama A, Kawakami H, Hashimoto K, Sasai Y (2015). Self-organization of polarized cerebellar tissue in 3D culture of human pluripotent stem cells. Cell Rep..

[16] Qian X (2016). Brain-region-specific organoids using mini-bioreactors for modeling ZIKV exposure. Cell.

[17] Nakagawa M (2008). Generation of induced pluripotent stem cells without Myc from mouse and human fibroblasts. Nat. Biotechnol..

[18] Onofre I (2016). Fibroblasts of Machado Joseph disease patients reveal autophagy impairment. Sci. Rep..

[19] de Chaumont F (2012). Icy: An open bioimage informatics platform for extended reproducible research. Nat. Methods.

[20] Pasca AM (2015). Functional cortical neurons and astrocytes from human pluripotent stem cells in 3D culture. Nat. Methods.

[21] Peng K (2020). Mechanisms underlying enhancement of spontaneous glutamate release by group I mGluRs at a central auditory synapse. J. Neurosci..

[22] Park D (2010). Nestin is required for the proper self-renewal of neural stem cells. Stem Cells.

[23] Di Lullo E, Kriegstein AR (2017). The use of brain organoids to investigate neural development and disease. Nat. Rev. Neurosci..

[24] Osumi N, Shinohara H, Numayama-Tsuruta K, Maekawa M (2008). Concise review: Pax6 transcription factor contributes to both embryonic and adult neurogenesis as a multifunctional regulator. Stem Cells.

[25] Raponi E (2007). S100B expression defines a state in which GFAP-expressing cells lose their neural stem cell potential and acquire a more mature developmental stage. Glia.

[26] Mendonca LS, Nobrega C, Hirai H, Kaspar BK, Pereira de Almeida L (2015). Transplantation of cerebellar neural stem cells improves motor coordination and neuropathology in Machado-Joseph disease mice. Brain J. Neurol..

[27] Imai T (2001). The neural RNA-binding protein Musashi1 translationally regulates mammalian numb gene expression by interacting with its mRNA. Mol. Cell Biol..

[28] Lasky JL, Wu H (2005). Notch signaling, brain development, and human disease. Pediatr. Res..

[29] Mohammad, L.W.J., Erickson, S., & Yang, G. *The Oxford Handbook of Neuronal Protein Synthesis* (ed. Sossin, W.S.). 1–31. (Oxford University Press, 2019).

[30] Gao Z (2009). Neurod1 is essential for the survival and maturation of adult-born neurons. Nat. Neurosci..

[31] Bathina S, Das UN (2015). Brain-derived neurotrophic factor and its clinical implications. Arch. Med. Sci..

[32] Bradford AB, McNutt PM (2015). Importance of being Nernst: Synaptic activity and functional relevance in stem cell-derived neurons. World J. Stem Cells.

[33] Wiedenmann B, Franke WW (1985). Identification and localization of synaptophysin, an integral membrane glycoprotein of Mr 38,000 characteristic of presynaptic vesicles. Cell.

[34] Renner M (2017). Self-organized developmental patterning and differentiation in cerebral organoids. EMBO J..

[35] Depla JA (2020). Cerebral organoids: A human model for AAV capsid selection and therapeutic transgene efficacy in the brain. Mol. Ther. Methods Clin. Dev..

[36] Yamada M (2014). Specification of spatial identities of cerebellar neuron progenitors by ptf1a and atoh1 for proper production of GABAergic and glutamatergic neurons. J. Neurosci..

[37] Goodlett, C.R. & Mittleman, G. Chapter 9—The cerebellum. in *Conn’s Translational Neuroscience*. 10.1016/B978-0-12-802381-5.00016-6 (Elsevier, 2017).

[38] Fink AJ (2006). Development of the deep cerebellar nuclei: Transcription factors and cell migration from the rhombic lip. J. Neurosci..

[39] Fujishima K, Horie R, Mochizuki A, Kengaku M (2012). Principles of branch dynamics governing shape characteristics of cerebellar Purkinje cell dendrites. Development.

[40] Yuan A, Rao MV, Nixon RA (2017). Neurofilaments and neurofilament proteins in health and disease. Cold Spring Harb. Perspect. Biol..

[41] Koch P (2011). Excitation-induced ataxin-3 aggregation in neurons from patients with Machado-Joseph disease. Nature.

[42] Williams AJ, Knutson TM, Colomer Gould VF, Paulson HL (2009). In vivo suppression of polyglutamine neurotoxicity by C-terminus of Hsp70-interacting protein (CHIP) supports an aggregation model of pathogenesis. Neurobiol. Dis..

[43] Amin ND, Pasca SP (2018). Building models of brain disorders with three-dimensional organoids. Neuron.

[44] Liu F (2019). Advances in cerebral organoid systems and their application in disease modeling. Neuroscience.

[45] Sidhaye J, Knoblich JA (2021). Brain organoids: An ensemble of bioassays to investigate human neurodevelopment and disease. Cell Death Differ..

[46] Mariani J (2015). FOXG1-dependent dysregulation of GABA/glutamate neuron differentiation in autism spectrum disorders. Cell.

[47] Yamaguchi M (2016). Neural stem cells and neuro/gliogenesis in the central nervous system: understanding the structural and functional plasticity of the developing, mature, and diseased brain. J. Physiol. Sci..

[48] Pollen AA (2019). Establishing cerebral organoids as models of human-specific brain evolution. Cell.

[49] Urban N, Guillemot F (2014). Neurogenesis in the embryonic and adult brain: same regulators, different roles. Front. Cell Neurosci..

[50] Engler A, Zhang R, Taylor V (2018). Notch and neurogenesis. Adv. Exp. Med. Biol..

[51] Goodman LJ (1996). Regulated release and polarized localization of brain-derived neurotrophic factor in hippocampal neurons. Mol. Cell Neurosci..

[52] Munoz E (2002). Intranuclear inclusions, neuronal loss and CAG mosaicism in two patients with Machado-Joseph disease. J. Neurol. Sci..

[53] Scherzed W (2012). Pathoanatomy of cerebellar degeneration in spinocerebellar ataxia type 2 (SCA2) and type 3 (SCA3). Cerebellum.

[54] Baumel Y, Jacobson GA, Cohen D (2009). Implications of functional anatomy on information processing in the deep cerebellar nuclei. Front. Cell Neurosci..

[55] Kikuchi T (2017). Human iPS cell-derived dopaminergic neurons function in a primate Parkinson's disease model. Nature.

[56] Nascimento-Ferreira I (2011). Overexpression of the autophagic beclin-1 protein clears mutant ataxin-3 and alleviates Machado-Joseph disease. Brain J. Neurol..

[57] Nobrega C (2019). Restoring brain cholesterol turnover improves autophagy and has therapeutic potential in mouse models of spinocerebellar ataxia. Acta Neuropathol..

[58] Li QF (2019). Neurofilament light chain is a promising serum biomarker in spinocerebellar ataxia type 3. Mol. Neurodegener..

[59] Wilke C (2020). Neurofilaments in spinocerebellar ataxia type 3: Blood biomarkers at the preataxic and ataxic stage in humans and mice. EMBO Mol. Med..

[60] Studer L, Vera E, Cornacchia D (2015). Programming and reprogramming cellular age in the era of induced pluripotency. Cell Stem Cell.

[61] Koch P (2015). Direct conversion provides old neurons from aged donor's skin. Cell Stem Cell.

[62] Kelava I, Lancaster MA (2016). Dishing out mini-brains: Current progress and future prospects in brain organoid research. Dev. Biol..

[63] Qian X, Song H, Ming GL (2019). Brain organoids: Advances, applications and challenges. Development.

[64] Mendonca LS (2019). Ibuprofen enhances synaptic function and neural progenitors proliferation markers and improves neuropathology and motor coordination in Machado-Joseph disease models. Hum. Mol. Genet..

