# Supplementary material for: Establishment and characterization of human pluripotent stem cells-derived brain organoids to model cerebellar diseases
Source: Sci Rep. 2022 Jul 22;12:12513. doi: 10.1038/s41598-022-16369-y (PMC9307606; doi:10.1038/s41598-022-16369-y)
Supplement: Supplementary file 1 — Supplementary Figures. [file 41598_2022_16369_MOESM1_ESM.pdf]

Establishment and characterization of human pluripotent stem cells-derived brain organoids to model cerebellar diseases

João Brás*[1,2], Daniel Henriques*[1,2], Ricardo Moreira*[1,2], Magda M. Santana[1,2,3], Rita Silva-Pedrosa[4,5], Diana Adão[1,2], Sandra Braz[1,2,3], Ana Rita Álvaro[1,2,3], Luís Pereira de Almeida$[1,2,6] and Liliana S. Mendonça$[1,2,3]

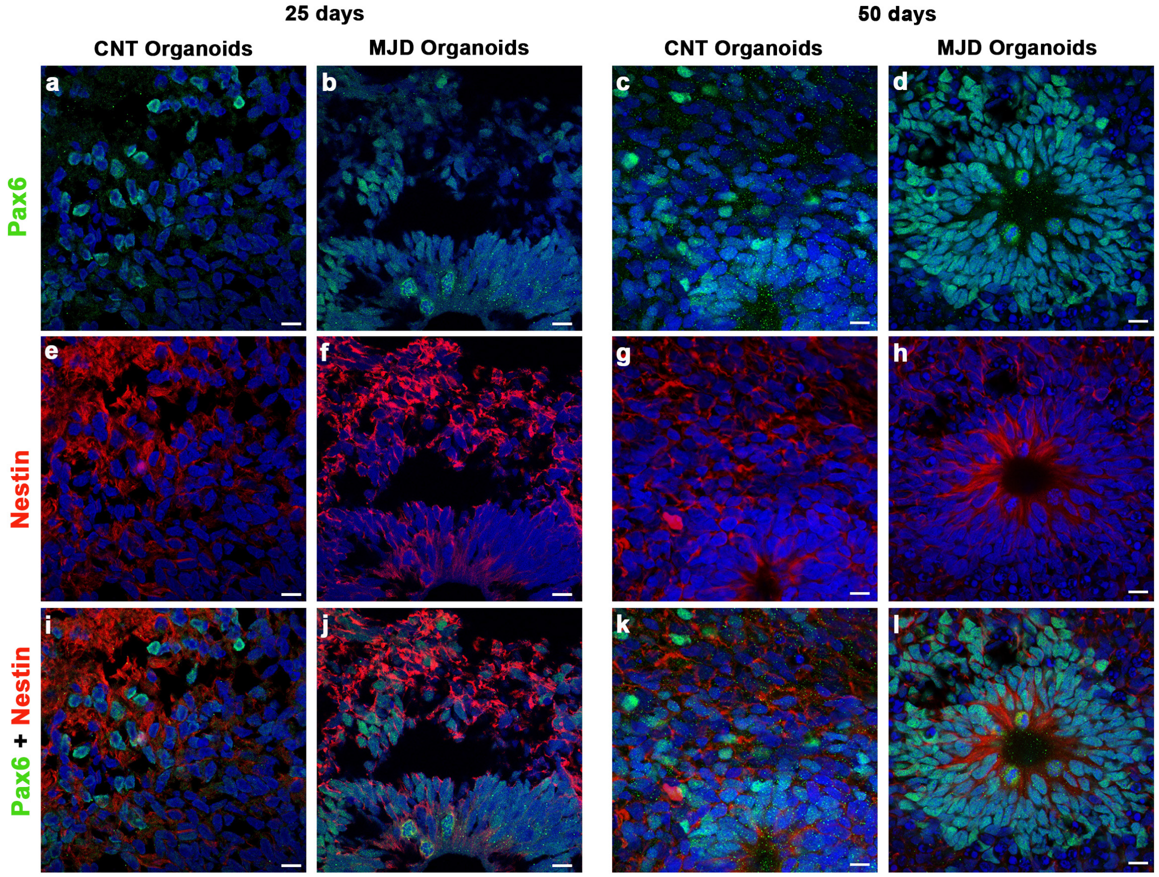

Figure Supplementary S1. Control and MJD brain organoids present Pax6- and Nestin-positive cells. Immunohistochemistry evaluation of CNT and MJD organoids with 25 and 50 days for **(a-d)** Pax6 (green), **(e-h)** Nestin (red), and **(i-l)** both (Pax6 and Nestin), demonstrating the presence of these markers, namely the expression of Pax6 in ventricular-like zones colocalizing with the nuclear marker DAPI. DAPI: blue, representative confocal microscopy images of 3 independent experiments, scale bars: 10 μm.

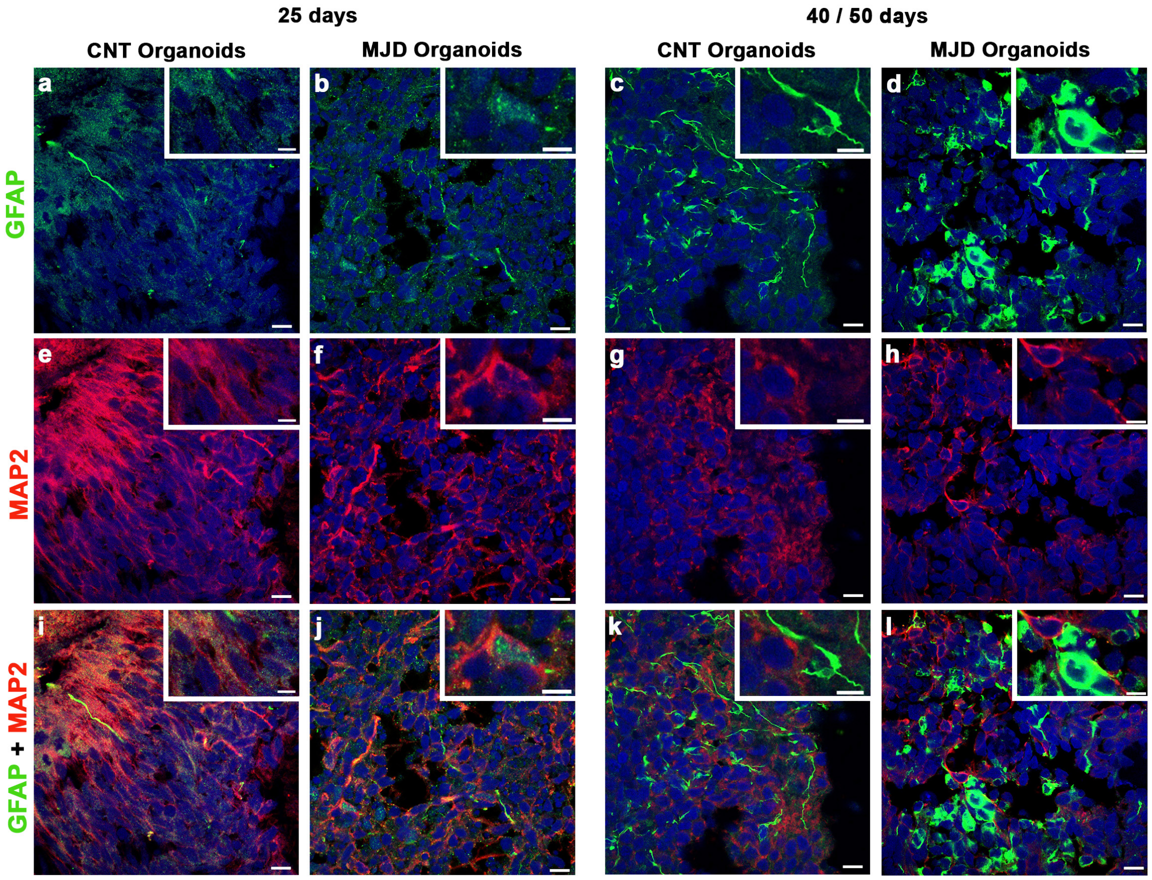

Figure Supplementary S2. Control and MJD brain organoids present glia and neurons. Immunohistochemistry evaluation of CNT and MJD organoids with 25 and 40/50 days for **(a-d)** GFAP (green), **(e-h)** MAP2 (red), and **(i-l)** both (GFAP and MAP2) expression. Data indicate that at day 25, neurons (MAP-2 positive cells) are already present **(e-f)** both in CNT and MJD organoids. Glial cells (GFAP-positive cells) are **(a-b)** very few at day 25 **(c-d)**, whereas at 40-50 days are abundantly detected. DAPI: blue, representative confocal microscopy images of 3 independent experiments. Upper inserts: higher magnification images showing GFAP (green) and MAP2 (red) positive cells. Scale bars: 10 μm and 5 μm in upper inserts.
